# Long-Standing Untreated or Inadequately Treated Seropositive Rheumatoid Arthritis in the Golden Age of Disease-Modifying Antirheumatic Drugs

**DOI:** 10.7759/cureus.78474

**Published:** 2025-02-04

**Authors:** Cristine K Arcilla, Gurjit S Kaeley, Myint Thway

**Affiliations:** 1 Department of Rheumatology, University of Florida College of Medicine – Jacksonville, Jacksonville, USA; 2 Department of Rheumatology, Baptist Health, Jacksonville, USA

**Keywords:** adherence, adherence to therapy, complication of rheumatoid arthritis, disease-modifying antirheumatic drugs, intervention in rheumatoid arthritis, medicine compliance, rheumatoid arthritis, rheumatoid severity

## Abstract

Rheumatoid arthritis (RA) is a chronic inflammatory disease presenting with articular and extra-articular manifestations that could be related to genetic and environmental factors. Seropositive RA is marked by the presence of autoantibodies, which are associated with worse prognoses and increased risk of extra-articular manifestations, including cardiovascular diseases, lung disorders, and malignancies.

Untreated RA can lead to substantial morbidity, reduced life expectancy, and poor quality of life. The development of disease-modifying antirheumatic drugs has paved the way for improving disease outcomes and reducing morbidity and complications related to disease. However, adherence to therapy remains a challenge, often influenced by drug intolerance, misconceptions, and negative beliefs regarding potential side effects and drug toxicity.

Here, we report three cases presenting with devastating complications due to long-standing untreated or inadequately treated seropositive RA. This emphasizes the huge disease burden and poor quality of life as part of the natural course and progression of untreated RA. Comprehensive patient education on the disease course, treatment benefits, and potential side effects is essential to enhance adherence and facilitate shared decision-making.

## Introduction

Rheumatoid arthritis (RA) is a systemic, progressive, chronic inflammatory disease causing significant joint pain, damage, and destruction in 80-85% of untreated patients along with multisystemic complications [1-4]. It has a prevalence of 1% worldwide and affects 1-2% of the Western population [3]. RA has a 3:1 female-to-male predominance, with disease onset in the fourth and fifth decades. Genetic and environmental factors contribute to the susceptibility and development of RA, with a higher association with smokers and strong family history [4,5]. Seropositive RA involves the detection of elevated RA-related autoantibodies such as rheumatoid factor (RF), antibodies to citrullinated protein antigens (ACPAs), antibodies to carbamylated proteins (anti-CarPs), and anti-acetylated proteins in one-half to one-third of the RA population [6]. These antibodies that cause autoimmunity are hypothesized to be triggered by mucosal inflammation from the oropharynx, lungs, and intestine, which is related to dysbiosis [4]. Seropositivity is a significant marker for developing RA and bone erosion. Seropositivity is also an indicator of a worse prognosis related to extra-articular involvement, such as cardiovascular mortality [6]. Almost half of RA patients could develop multisystemic involvement. These extra-articular manifestations and RA complications could range from ischemic heart disease, interstitial lung disease, osteoporosis, metabolic syndrome, ocular inflammation (episcleritis, scleritis, etc.), malignancies (non-Hodgkin lymphoma, etc.), hematologic abnormalities (Felty syndrome, etc.), and cervical myelopathy related to atlantoaxial and subaxial subluxation, among others [5-10]. One study summarized the incidence and severity of extra-articular complications of RA based on geographical variations, supporting further the effects of genetic and environmental factors in the overall disease course of RA in every individual [10]. Dysregulated activation of immune cells (T and B-cells, macrophages, fibroblasts), pro-inflammatory chemokines and cytokines (tumor necrosis factor-α, IL-1, IL-6, etc.), and local growth factors directly causing organ inflammation is one of the possible underlying immunopathological mechanisms of RA [5-7].

If inadequately or not treated, especially in the early stages, RA can cause a substantial personal and socioeconomic burden, impacting productivity and function [1,7-9]. The prognosis of RA is comparable to that of cardiovascular mortality and terminal-stage cancer, with life expectancy reduced by 3-12 years compared to the general population [1,2,5,7,9]. Additionally, RA can lead to debilitating, permanent joint deformities that result in a poor quality of life, significant morbidity, and psychological distress [9]. A more proactive approach involves the earlier identification and diagnosis of RA, coupled with aggressive treat-to-target strategies aimed at achieving clinical remission or low disease activity. Recent advances in therapy, including biologic agents targeting the pathophysiologic and immunologic mechanisms of RA, have significantly improved disease outcomes [1,8,10-12]. Disease-modifying antirheumatic drugs (DMARDs) are used for a prolonged period to control and modify the natural course of the disease, including radiographic progression and extra-articular comorbidities [13]. However, the introduction of multiple DMARDs and their combinations has also brought about concerns, misconceptions, and negative beliefs regarding potential side effects and drug toxicity, which can limit the utility of DMARDs in some patients [14]. Several studies have reported non-adherence to RA therapy as a factor contributing to poor clinical outcomes due to several factors including drug intolerance [2,15,16]. Here, we report a case series of three patients who presented with devastating complications due to long-standing untreated or inadequately treated seropositive RA.

This article was previously presented as a meeting abstract poster at the 2024 Florida Society of Rheumatology Annual Meeting on July 13, 2024.

## Case presentation

Case 1

**Figure 1:**
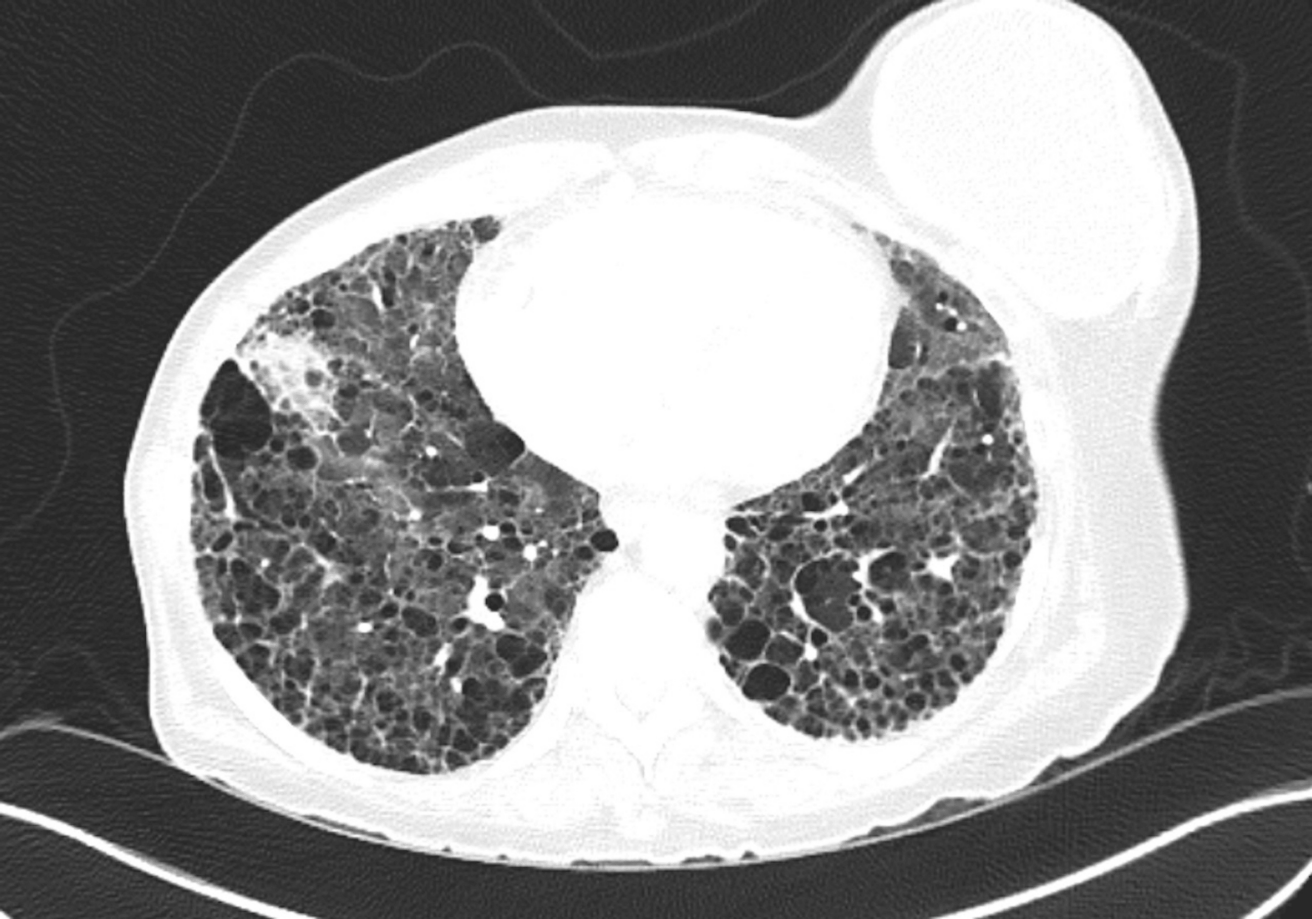
Chest CT showed usual interstitial pneumonia with new ground-glass opacities in all lobes.

**Figure 2:**
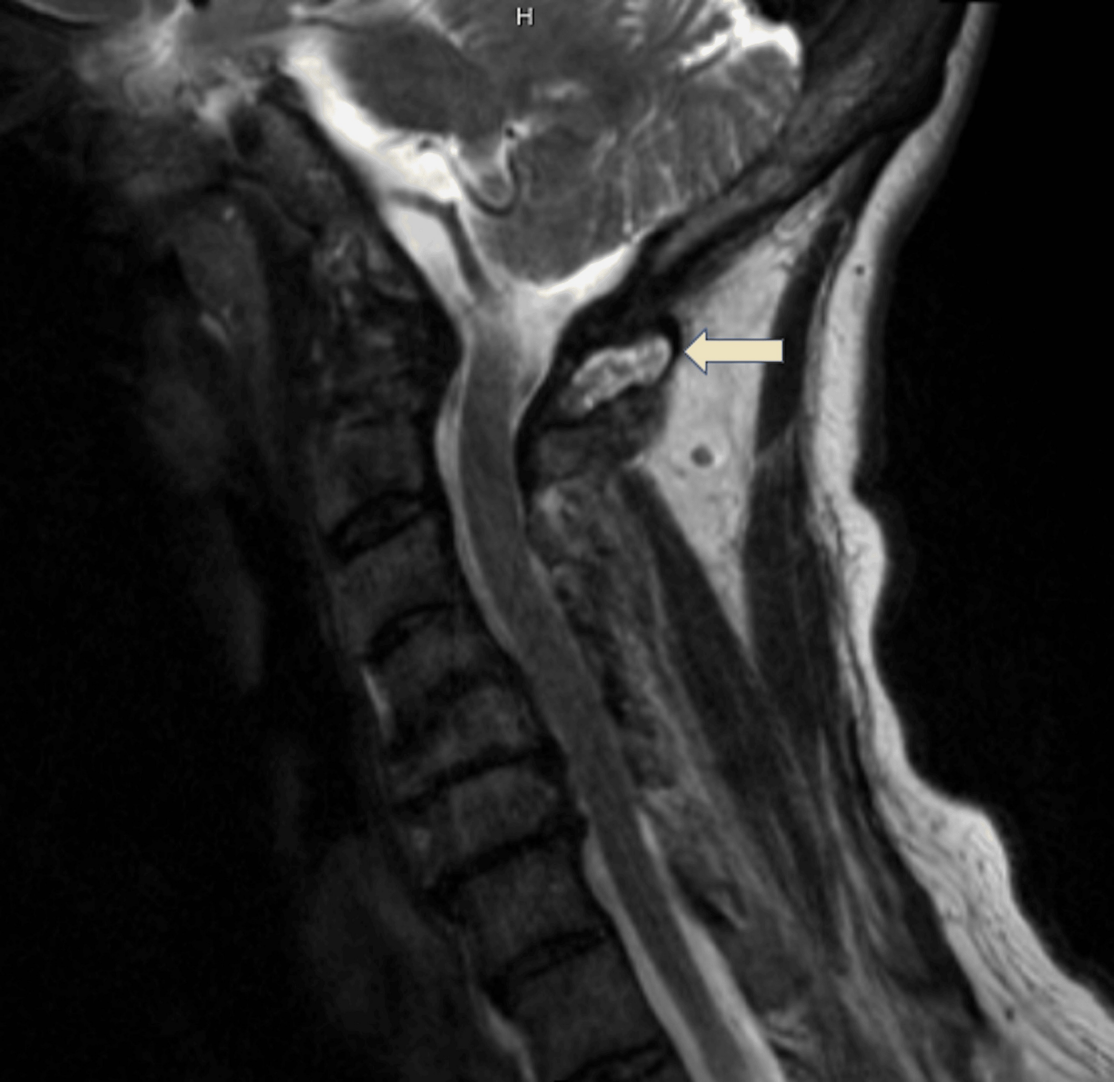
MRI of the cervical spine revealing extensive severe first and second cervical vertebrae (C1-C2) erosions with retrodental pannus with effusion and multilevel stenosis.

A 60-year-old female with a medical history of pulmonary emphysema, seropositive erosive RA for more than 10 years, and interstitial lung disease (ILD) presented to the hospital for progressively worsened shortness of breath and significant hypoxia. She was previously on adalimumab and subsequently etanercept for a few months after diagnosis, which she stopped believing these have worsened her symptoms. She declined therapy for her ILD when later found out. She reported occasional burning sensation on her feet but denied urinary or fecal incontinence. Physical examination was remarkable for acute respiratory distress on high-flow nasal oxygenation, diffuse rhonchi and crackles, multiple rheumatoid nodules, severe deformities, and synovitis of major joints with limited range of motion. Cardiac workup was notable for elevated brain natriuretic peptide and flat troponin elevation. Transthoracic echocardiography revealed septal flattening suggestive of right ventricle volume overload concerning for pulmonary hypertension. Chest CT showed usual interstitial pneumonia (UIP) with new ground-glass opacities in all lobes and diffuse lymphadenopathy (Figure 1). A previous cervical MRI three months ago revealed extensive severe first and second cervical vertebrae (C1-C2) erosions with retrodental pannus with effusion and multilevel stenosis (Figure 2). She was at high risk of cervical myelopathy, quadriplegia, and even death with any procedure requiring cervical manipulation in the course of worsening respiratory failure.

Case 2

**Figure 3:**
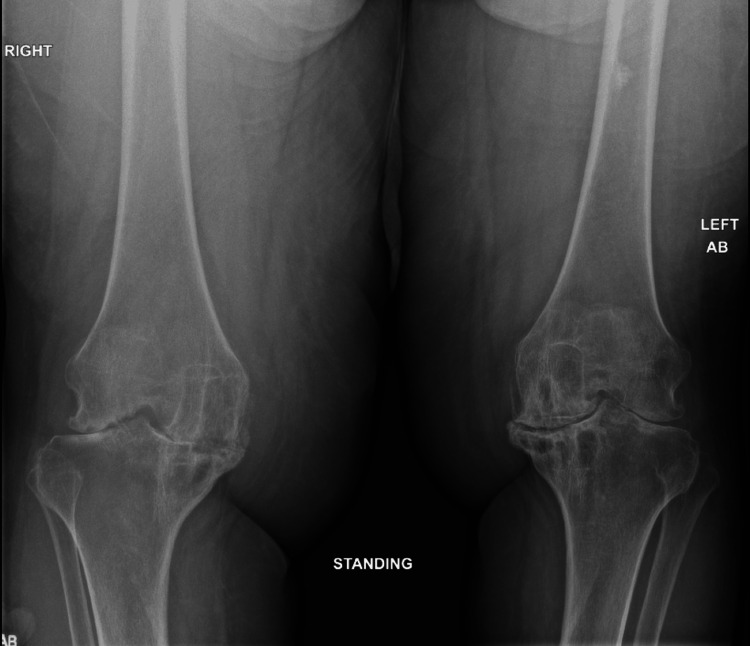
Knee radiography showing severe tricompartmental osteoarthritis bilaterally with symmetrical joint narrowing.

**Figure 4:**
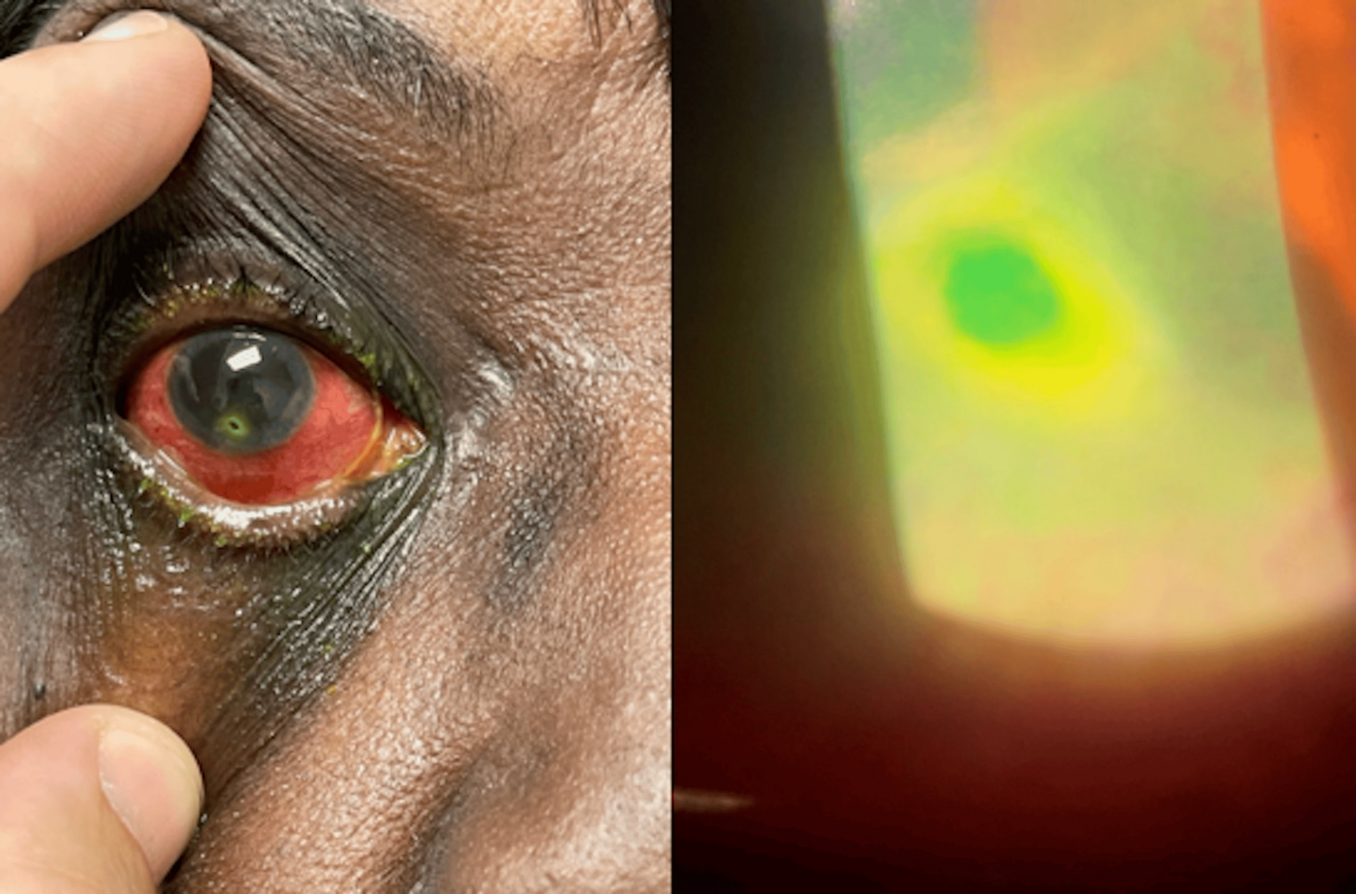
Physical examination and slit-lamp examination showing a conjunctival injection with greater than 80% thinning of a peripheral ulcer/melt without infiltrate with fluorescein staining over a small area of corneal thinning on the right eye. Courtesy of Dr. Braeden Moody.

A 69-year-old female with a medical history of long-standing seropositive RA for more than 30 years and perforated corneal ulcer of the left eye secondary to peripheral ulcerative keratitis presented to the hospital for progressive right eye pain, redness, and photosensitivity with visual loss for the past three days. She was previously on methotrexate, hydroxychloroquine, and steroids for a few weeks upon diagnosis, which she stopped due to fear of side effects. Physical examination was remarkable for bilateral conjunctival injection and corneal clouding with discharge, multiple rheumatoid nodules, severe joint deformity, including ulnar deviation and subluxation, and several severe synovitis (Figure 3). Slit-lamp examination showed greater than 80% thinning of a peripheral ulcer/melt without infiltrate with fluorescein staining over a small area of corneal thinning on the right eye and diffuse stromal scarring inferiorly on the left eye (Figure 4). High-sensitivity C-reactive protein (33 mg/L) and sedimentation rate (81 mm/hour) were elevated. Methylprednisolone at a dose of 1 g/day for three days was initiated and transitioned to a prednisone 1 mg/kg taper upon discharge. A follow-up outpatient ophthalmology examination showed a perforated ulcer of the right eye, requiring glue and placement of a bandage contact lens. A plan for infliximab infusions was contemplated, and she was agreeable and adherent to care after multiple careful discussions of benefits over risks.

Case 3

**Figure 5:**
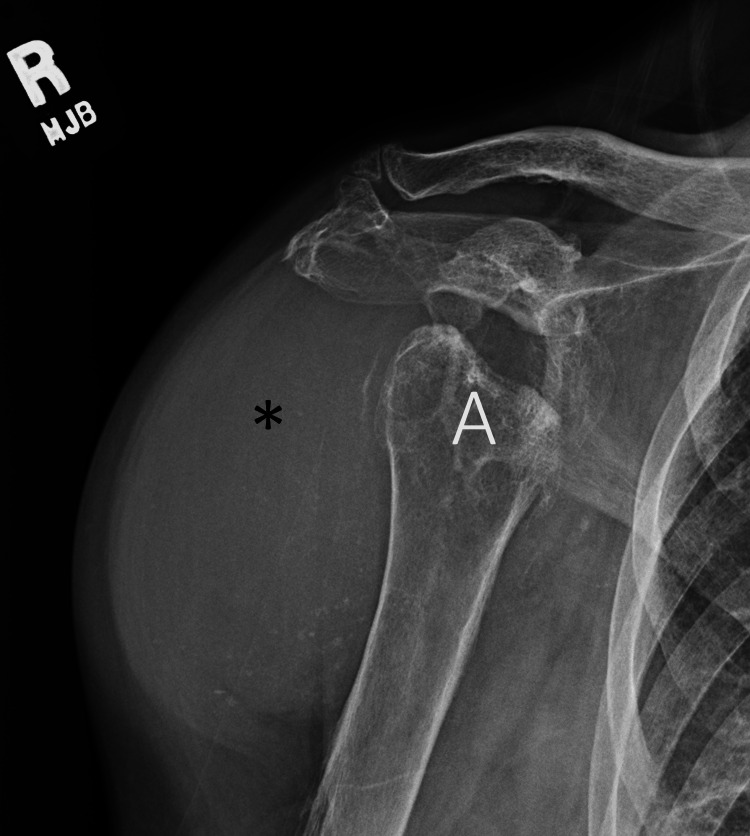
Right shoulder radiography in external rotation showing remnants of the eroded humeral head (A), with marked shoulder peri-articular swelling (asterisk).

**Figure 6:**
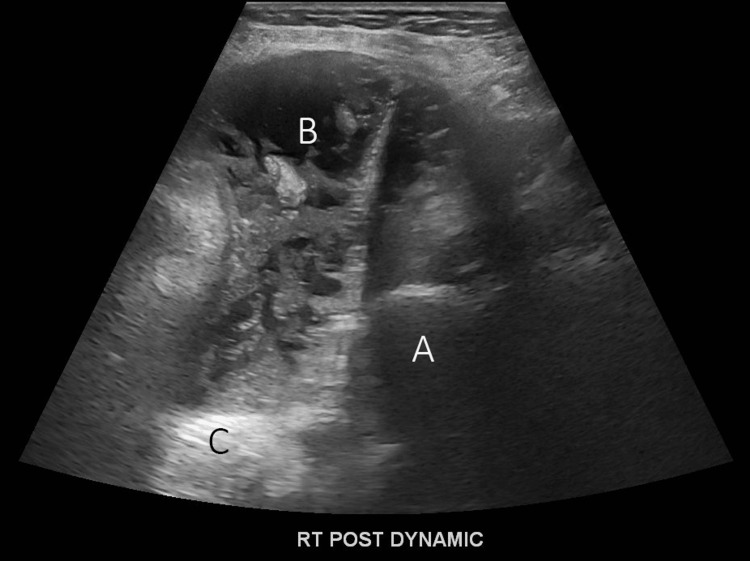
Ultrasound of the posterior view of the shoulder showing remnant of the humeral head (A), surrounded by marked intracapsular tissue and fluid (B), and the adjoining scapula (C).

A 73-year-old female with a medical history of long-standing seropositive RA of more than 20 years, osteopenia, ILD-UIP, and paroxysmal atrial fibrillation on anticoagulation presented to the clinic for severe right shoulder pain and swelling. She was lost to follow-up for four years and took hydroxychloroquine and leflunomide inconsistently in the past. She used a wheelchair for ambulation and needed moderate assistance with performing activities of daily living, including self-care. Physical examination was notable for severe fixed flexor deformities of elbows and hands, bilateral wrist fusion, and severe bilateral shoulder synovitis and fluctuance, right greater than left. Right shoulder radiography revealed collapse and erosive changes of the humeral head and glenoid with prominent soft tissue swelling in the lateral aspect of the shoulder (Figure 5). Right shoulder ultrasound showed marked destruction of the humerus along with multiple tendon tears and large effusion (Figure 6). Initial aspiration of the joint revealed straw-colored cloudy fluid. Synovial fluid analysis was investigated for infection.

## Discussion

We present three patients with rare, severe clinical presentations of RA. This emphasizes the huge disease burden and impact of RA on patients’ quality of life, including a high risk of morbidity and mortality, permanent vision loss, and other severe organ manifestations as part of the natural course and progression of RA without treatment with DMARDs [3,17]. Overall, 1-2% of the global adult population is estimated to be affected by RA, with 1.5 million adults in North America [18]. One study reported hypertension, cardiovascular diseases (specifically coronary artery disease), and diabetes as the most prevalent comorbidities associated with RA [19]. In another study, the most common multisystemic complications of RA over 10-year follow-up included rheumatoid nodules, pulmonary fibrosis, sicca syndrome, and anemia of chronic disease [10]. Corneal melts, rheumatoid nodules, atlantoaxial subluxation, and vasculitis are observed to be less prevalent nowadays [20].

Wang et al. pointed to the continuous increase in the prevalence of RA-ILD from 1.7% to 71.6% with a high mortality rate of 13% and median survival of 3-7 years [21]. Indians and African Americans have increased predisposition and earlier development of RA-ILD, respectively, compared to other ethnicities. Male sex (with a two-fold increased risk), advanced age, smoking, later age of disease onset, positive RA serologies such as RF and ACPAs, elevated inflammatory marker, longer disease duration, pulmonary comorbidities, moderate to high articular disease activity (with a two-fold increased risk than low activity or remission), rheumatoid nodules, and steroid use were considered predictors of RA-ILD [22]. On the other hand, biologic DMARDs showed a protective factor from the development of RA-ILD. Early stages of RA-ILD are usually subclinical and asymptomatic, which may cause a delay in clinical diagnosis until the development of irreversible pulmonary fibrosis with overt and severe clinical symptoms. Hence, utilization of high-resolution CT is recommended by the American College of Rheumatology and American College of Chest Physicians as a non-invasive method for baseline screening, diagnosis, and monitoring among RA-ILD patients along with other systemic autoimmune rheumatic diseases that have higher risks for ILD [23]. RA-ILD could be characterized based on histologic and/or imaging features with UIP as the most common and usually most aggressive subtype. Aggressive control of disease activity through a treat-to-target strategy is crucial in the prevention and delay of progression of RA-ILD complications. Immunomodulatory and antifibrotic drugs may also treat RA-ILD.

Erosive pannus formation could involve vertebral articulation such as in the first and second cervical vertebrae, which may lead to the destruction of the spinous process and apophyses, inflammatory discitis, ligamentous laxity (e.g., transverse ligament), and subsequent subluxation and fusion [20]. Overall, 43-86% of patients with RA have cervical spine involvement showing as radiographic cervical spine involvement, while anterior atlantoaxial subluxation is the most common complication with a prevalence rate of 10-55%. Higher risks for cervical manifestations were described in patients with peripheral joint erosions, chronic corticosteroid use, higher disease activity, elevated inflammatory markers, and DMARD failure. Involvement of the cervical spine can occur as early as within two years of initial RA diagnosis and is usually asymptomatic or could have associated neck pain [18]. Neurological compromise such as vertebrobasilar insufficiency or brainstem and spinal cord compression could be part of later sequelae of atlantoaxial instability and could impose significant morbidity and mortality on patients with RA when left untreated. With the advent of better disease control with DMARDs, the prevalence of anterior cervical subluxation has decreased to 27%. Continuous treatment with conventional, biologic, and combination DMARDs for more than two years was observed to prevent anterior atlantoaxial subluxation [20].

Peripheral ulcerative keratitis (PUK) is reported as the initial manifestation in half of cases with collagen vascular disease with a prevalence of 1-2% in RA [24]. It is caused by stromal inflammation and collagen degradation causing juxtalimbal corneal ulceration with sectorial thinning and could involve adjacent conjunctival, episcleral, and scleral inflammation. Bilateral involvement is reported in 40% of cases. Necrotizing scleritis is also observed in severe RA cases, which could further complicate to corneal perforation and visual loss. Higher risks for PUK are reported in patients with long RA duration, inconsistent treatment, keratoconjunctivitis sicca, erosive arthritis, and positive serologies (RF and ACPA) but PUK may also precede the systemic presentation of RA. Untreated RA patients with PUK or necrotizing scleritis have a 50% mortality rate in 10 years. Aggressive systemic treatment with timely regular eye surveillance and corresponding early and urgent management upon detection with combination therapy of topical and systemic steroid, biologic DMARD, and immunosuppressive agents including calcineurin inhibitors are critical for preventing the progression of eye complications such as vasculitis and remission of ulcer [25].

More severe erosions are found greater in patients with positive RA serologies, high disease activity, and early presence of bony erosions [26]. Early DMARD initiation is crucial for attaining sustained remission in the first six months with the prevention of irreversible joint damage. Early detection of erosions through better utilization of imaging is essential to determine a more intensive treat-to-target approach. Conventional radiography is limited in the detection of the early stages of the disease. MRI has the highest sensitivity for the detection of early erosions and even pre-erosive characteristics, such as bone marrow edema, synovitis, and tendinous and ligamentous abnormalities but is expensive for routine follow-up [27]. Ultrasound has been utilized for evaluation of early signs of synovitis and erosions and follow-up following therapy greater than radiography. The most discriminating location for erosions for ultrasound in one study was found in the fifth metatarsophalangeal joint (MTP5). In addition, synovitis in the wrists, hands (second metacarpophalangeal joint), and feet (MTP5) is seen in the early stages of the disease and joint damage. On the other hand, synovitis in medium and large joints of shoulders and hips, knees, temporomandibular joint, elbows, and ankles is noted in the latter course of RA and correlates with severe disease activity.

Therefore, multiple treatment targets for RA aim to slow disease progression, achieve complete disease remission, minimize systemic complications and drug side effects, and improve quality of life [5,9]. According to the 2021 American College of Rheumatology guidelines, methotrexate, a conventional synthetic DMARD (csDMARD), is considered the gold standard for RA treatment due to its efficacy, relative safety, and cost-effectiveness. Other csDMARDs, including hydroxychloroquine, sulfasalazine, and leflunomide, also offer significant benefits to RA patients, including cardiovascular protection [6]. Since the introduction of biological DMARDs (bDMARDs) in 2000, such as anti-tumor necrosis factor agents, and later target synthetic DMARDs (tsDMARDs), such as Janus kinase inhibitors, there have been dramatic improvements in disease outcomes [3,5,9,17]. Each DMARD has its mechanism and site of action, contributing to its unique cellular properties and clinical profile. Monoclonal antibodies or receptor proteins inhibit soluble or cell surface molecules, such as cytokines, cytokine receptors, or other cell membrane antigens. In contrast, tsDMARDs are oral small molecules that inhibit cytokine signaling and intracellular signal transduction pathways.

However, DMARDs also present potential adverse events, including bone marrow suppression, elevated liver enzymes, retinal toxicity, opportunistic infections and latent reactivation, cancer, and demyelinating diseases, among others. One study found that non-adherence to DMARDs occurred in 10% of the population, attributed to factors such as medication-related concerns and intolerance, forgetfulness, and RA disease activity on remission [14,16]. Other studies noted that a perceived lack of therapeutic efficacy, despite ongoing therapy, contributed to non-adherence [2]. According to a cross-sectional study, lower socioeconomic status and significant comorbidities were associated with decreased medication adherence [14]. Conversely, high adherence was linked to effective patient-physician collaboration and understanding of the illness.

Therefore, treatment decisions for RA should be tailored to each individual’s clinical characteristics, comorbidities, preferences, and tolerance. Patient counseling on the benefits and risks of available treatment, alongside potential mitigation strategies for side effects and understanding the natural chronic progressive course of RA with possible debilitating systemic complications, if untreated, should be emphasized in each patient encounter. Risk stratification for RA screening, monitoring, and prognostication can be essential strategies to increase patient adherence to therapy. Identification of individual risk factors for extra-articular complications including modifiable and non-modifiable factors is crucial in every counseling and might be employed as objective evidence to patients for their predisposition to long-term complications of RA. Incorporating discussions regarding lifestyle modifications such as smoking cessation and weight loss, control of other medical comorbidities, and constant surveillance of the progression of RA are essential therapeutic strategies to improve prognosis.

A randomized control trial showed significant improvements in pain and other RA symptoms through a patient education program [28]. Patient education consisted of an explanation of RA, clinical presentation, available treatment, and lifestyle management. Individualized or grouped behavioral and psychosocial counseling from four weeks to six months improved patient-reported outcomes with RA [29,30]. These interventions serve as an effective adjunct to standard medical therapy as we build trust with our patients. This holistic approach might also include careful discussions of subjective individual experiences of other patients aside from objective discussions from current, updated publications and ongoing trials. In the second case, utilizing behavioral counseling helped in ensuring an informed, shared decision-making for the patient while enhancing her understanding of her diagnosis. This subsequently encouraged her to start anti-tumor necrosis factor therapy with good adherence seeing the significant change in her vision. Hence, it is imperative to include an individualized and personalized approach to every patient, understanding that each patient has unique needs and experiences that contribute to their decision-making regarding their overall aspect of care. Understanding the importance of behavioral counseling could pave the way for the development and involvement of multidisciplinary teams in every institution, if not currently available, dedicated as patients’ support and awareness groups. This team could involve the primary care provider, rheumatologist, pulmonologist, ophthalmologist, orthopedic surgeon, pharmacist, allied medical professionals, and behavioral therapists, among others. Advocacy programs through the local community and institutions, and even through national and international efforts through the aid of rheumatology societies and social media, are also important sectors that could be tapped for facilitating patient awareness and advocacy for RA.

## Conclusions

Treatment decisions for RA should be tailored to each individual’s clinical characteristics, comorbidities, preferences, and tolerance. Patient counseling on the benefits and risks of treatment, alongside potential mitigation strategies for side effects and understanding the natural course of RA if untreated, remains central to RA management. This approach ensures informed, shared decision-making and enhances patients’ understanding of their diagnosis, adherence to therapy, and prevention of long-term systemic consequences.
